# Union Med. Society of Northern Indiana

**Published:** 1847-12

**Authors:** 


### UNION MEDICAL SOCIETY OF NORTHERN INDIANA.

We received from Dr. P. Henkle, Corresponding Secretary of this spirited Association, an abstract of the proceedings of its meeting, at Benton, held on the 2d Tuesday of November last, too late for their insertion in the proper place.

Dr. W. J. Matchett read a paper on the subject of the use and abuse of calomel, which was ordered to be sent to this Journal for publication. Dr. M. M. Latta was appointed delegate to the National Medical Association. The next meeting is to be held at Goshen, on the second Tuesday of May next. E.

